# Robotic central pancreatectomy in pediatric patients: a systematic review and technical innovation of externalized duct stent reconstruction

**DOI:** 10.1007/s11701-026-03424-3

**Published:** 2026-04-22

**Authors:** Alice Cattelani, Martina Guerra, Alessio Morandi, Alessandro Giardino, Isabella Frigerio, Paolo Regi, Filippo Scopelliti, Erica Pizzocaro, Elisa Bannone, Giovanni Butturini

**Affiliations:** 1 HPB Unit, General Surgery Department, Pederzoli Hospital, Peschiera, 37019 Italy; 2 Department of Human Sciences, University of Verona, Lungadige Porta Vittoria, Verona 17 - 37129 Italy

**Keywords:** Robotic surgical procedures, Pancreatectomy, Pediatric surgery, Pancreaticojejunostomy, Stents, Pancreatic fistula

## Abstract

**Background:**

Robotic pancreatic surgery in children remains rarely reported. Central pancreatectomy (CP) offers a parenchyma-sparing option for benign or low-grade malignant tumors. Adult data suggest externalized duct stents may mitigate postoperative pancreatic fistula (POPF), but pediatric evidence is lacking.

**Methods:**

A systematic search of MEDLINE, Embase and the Cochrane Library (November 2025) identified reports of robotic pancreatic resection in pediatric patients. Data on pathology and perioperative outcomes were extracted. We additionally provide a technical description of a reproducible surgical approach applied in two consecutive pediatric patients undergoing robotic CP with Roux-en-Y duct-to-mucosa pancreaticojejunostomy (PJ) and externalized pancreatic duct stent at a high-volume pancreatic center. A detailed video documents the step-by-step technique. Perioperative and 6-month outcomes are reported.

**Results:**

Ten case reports were included. Indications were solid pseudopapillary tumor (SPT) (n = 7), insulinoma (n = 2), and pancreatic neuroendocrine tumor (NET) (n = 1). Procedures comprised distal pancreatectomy (DP) (n = 5), pancreatoduodenectomy (PD) (n = 2), CP (n = 1), and enucleation (n = 2). PJ was used in all reconstructive procedures, and no study reported stent placement. POPF occurred in 1 patient after CP; other complications were infrequent. The described technique was applied in two pediatric: one developed biochemical leak (BL) and one grade B POPF, managed conservatively. At 6 months, pancreatic function was preserved with no recurrence.

**Conclusion:**

Robotic CP with PJ and externalized pancreatic duct stent is technically feasible in selected pediatric patients. However, given the limited evidence and lack of comparative data, no conclusions can be drawn regarding effectiveness. These findings are hypothesis-generating and require validation in multicenter studies.

**Supplementary Information:**

The online version contains supplementary material available at 10.1007/s11701-026-03424-3.

## Introduction

Pediatric pancreatic disease differs substantially from that in adults. Resection is most often indicated for benign or low-grade malignant neoplasms — such as solid pseudopapillary tumors (SPT)—for which long-term survival is expected [1]. Accordingly, surgical strategy should prioritize preservation of pancreatic and splenic function to minimize lifelong risks of diabetes, malabsorption, and infection, including overwhelming post-splenectomy infection (OPSI).

For lesions arising in the pancreatic neck or proximal body, central pancreatectomy (CP) is a parenchyma-sparing option that preserves the pancreatic head, distal pancreas, and spleen. This procedure maximizes preservation of endocrine and exocrine pancreatic function, which are less preserved after distal pancreatectomy (DP) [2–4]. Splenic preservation is equally critical, as it prevents the risk of OPSI and reduces susceptibility to arterial and venous thrombosis [5]. 

Robotic platforms may further enhance this conservative intent in children and adolescents. Three-dimensional visualization, enhanced dexterity, and wristed instrumentation can facilitate meticulous dissection and precise reconstruction within small operative fields, potentially reducing tissue trauma and incision length [6]. Given the fragile anatomical structures in pediatric surgery, the proportional benefit of combining a robotic-assisted approach with parenchyma-sparing resection may be even greater than in adults. However, although robotic pancreatic resections are well described in adults [7, 8], pediatric experience remains scarcely reported [9]. 

Despite the theoretical advantages of CP, its adoption has been tempered by higher rates of postoperative pancreatic fistula (POPF) possibly arising from both proximal and distal pancreatic stumps [10]. In this setting, an externalized pancreatic duct stent (transanastomotic endo-Wirsung stent) has been shown to decrease POPF severity, providing additional safety during the critical healing phase [11, 12]. Despite its documented role in adult open surgery, its adoption during robotic CP—particularly in pediatric patients—remains unexplored.

The present study aims to (i) systematically review the available literature on robotic pancreatectomy in children and adolescents, and (ii) provide a detailed technical description of a reproducible surgical approach consisting of robotic CP with Roux-en-Y PJ and externalized pancreatic duct stent placement, evaluating its feasibility and short- and mid-term outcomes. Given that current evidence is limited to isolated case reports, these findings should be interpreted as descriptive and hypothesis-generating rather than evidence of clinical effectiveness.

## Materials and methods

### Study design and registration

This systematic review has been registered with the National Institute for Health Research (NIHR) in PROSPERO (ID 1218881). This review was conducted and reported in accordance with the Preferred Reporting Items for Systematic Reviews (PRISMA) statement (Supplementary Sect. 1) [13]. In addition, we reported a technical description of two consecutive robotic CP with Roux-en-Y PJ and externalized pancreatic duct stent placement performed in pediatric patients at a single high-volume pancreatic surgery center (Pederzoli Hospital, Peschiera del Garda, Italy).

### Search strategy

A comprehensive systematic search in MEDLINE (via PubMed), Embase and the Cochrane Library was conducted in November 2025 to identify studies reporting robotic pancreatic resections in pediatric patients. Full search strategies for each database are provided in Supplementary Sect.  2. References lists of all eligible reports were hand-screened to identify additional studies. Only English-language, human studies were included.

### Study eligibility criteria

Studies were eligible if they met the following criteria:

- Population: children and adolescents (< 18 years) undergoing pancreatic resection.
- Intervention: robotic pancreatectomy of any type (DP, CP, total pancreatectomy, or pancreatoduodenectomy (PD)).
- Outcomes: perioperative outcomes, pancreatic anastomosis type, use of externalized pancreatic duct stents, postoperative morbidity, and functional outcomes (endocrine or exocrine).
- Study design: Randomized controlled trials (RCTs), case series, retrospective and prospective observational studies, case reports published in peer-reviewed journals.

Exclusion criteria were:

- Studies including only adult patients (≥ 18 years).
- Non-robotic procedures (open or purely laparoscopic).
- Reviews, editorials, commentaries, conference abstracts lacking sufficient clinical details.
- Animal studies, and non-English language publications.

### Study selection and data management

Records were imported into Ryyan and duplicates were removed both automatically and manually. Two reviewers (AC and AM) independently screened titles and abstracts, followed by full-text assessment against the eligibility criteria. Disagreements were resolved by consensus or third reviewer adjudication when necessary.

### Data extraction

The same reviewer independently extracted data from all included studies using a standardized data collection form. Extracted variables included: year of publication, country, patient demographics (i.e.: age, sex), indication for surgery, pancreatectomy type, reconstruction technique (PJ vs. PG), use of externalized pancreatic duct stents, operative time, estimated blood loss, length of hospital stay, postoperative morbidity, and follow-up outcomes including exocrine and endocrine pancreatic function. Postoperative complications were graded using the Clavien–Dindo classification [14] POPF, delayed gastric emptying (DGE), and post pancreatectomy haemorrhage (PPH) were defined and graded according to the International Study Group for Pancreatic Surgery (ISGPS) criteria [15–17]. 

### Risk of bias assessment

Given the anticipated predominance of case reports and small case series, study-level quality within the studies was appraised using the Joanna Briggs Institute (JBI) critical appraisal tools for case reports. Assessments were performed independently by two reviewers with consensus used for resolve disagreements. Certainty-of-evidence (GRADE) assessment was not applied due to the descriptive, non-comparative nature of evidence.

### Synthesis methods

Given the clinical and methodological heterogeneity (including patient age, indication for surgery, resection and reconstruction type), a meta-analysis was not planned. Findings were synthesized narratively, with tabulation of study characteristics and outcomes. All data are presented in a purely descriptive manner, and no formal pooling or quantitative aggregation was performed given the small sample size and heterogeneity of included studies. No formal assessment of small-study or publication bias was undertaken owing to the small number and design of included studies.

### Clinical cases

In addition to the systematic review, we report our technical description of two consecutive pediatric patients who underwent robotic CP with Roux-en-Y PJ and externalized pancreatic duct stent. For each case, demographics, indication, operative details, postoperative complications (graded per Clavien–Dindo classification and ISGPS criteria) [14–17], and length of hospital stay were prospectively collected. Mid-term follow-up at 6 months evaluated endocrine and exocrine pancreatic function, and early oncological outcomes. Clinical data were collected prospectively and entered into the institutionally approved “Pancreatic Disease Registry” (Ethics Committee Approval Number 31615–20/01/2020). Furthermore, written informed consent was secured from the patients’ legal guardians.

## Results

### Study selection

**Fig. 1:**
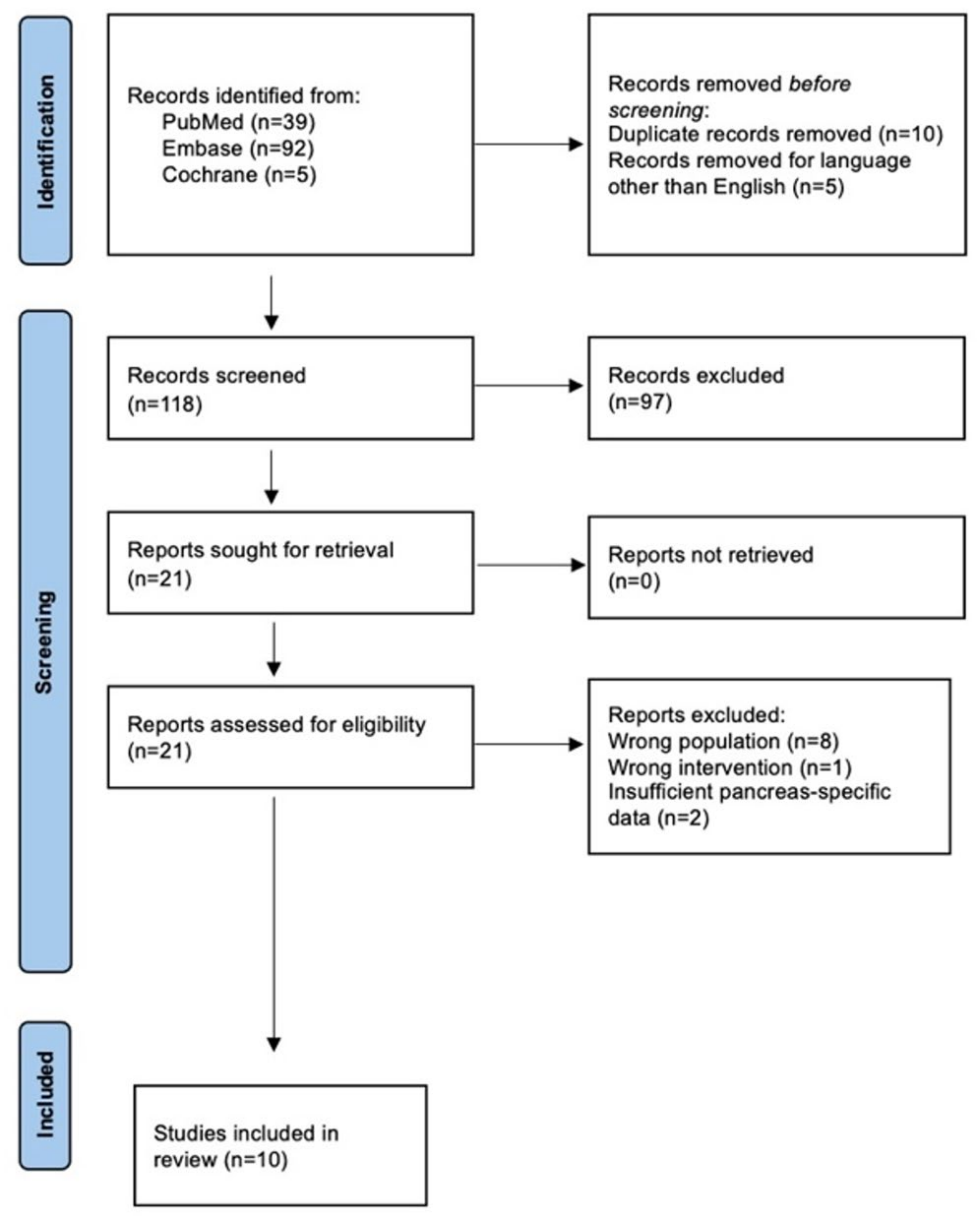
PRISMA Flowchart for Search Strategy

The database search yielded 133 records. After removal of duplicates and titles and abstracts screening, 21 full texts were assessed for eligibility, and 10 studies were included in the systematic review [18–28]. The PRISMA flow diagram detailing the selection process is shown in Fig. 1. The most frequent reasons for exclusion at full-text review resulted from adult dominant cohorts without separable pediatric data and non-robotic procedures.

### Characteristics and outcomes of included studies

All ten included studies were single case reports, published between 2017 and 2025, reporting a cumulative total of 10 patients. Main characteristics and outcomes are summarized in Table 1. Patients’ ages ranged from 9 to 17 years (median 11 years; interquartile range [IQR] 10–16). The most common indication for surgery was SPT (*n* = 7) [18, 21–23, 26–28], followed by insulinoma (*n* = 2) [19, 24], and non-functioning pancreatic neuroendocrine tumor (pNET, *n* = 1) [20]. The most frequent procedure was DP (*n* = 5) [20, 22, 24, 27, 28], including two spleen-preserving DP [24, 28], followed by PD (*n* = 2) and enucleation (*n* = 2) [19, 21, 23, 26]. Only one CP was reported [18].

**Table 1:** Characteristics and outcomes of studies included in the systematic review on robotic pancreatectomy in children

Author (year)	Country	Study type	No.	Age (years)	Indication	Type of pancreatectomy	Reconstruction type	Stent Used	Operative time (min)	EBL (ml)	Complications (POPF)	LOS (days)
Hu (2017) [24]	China	Case report	1	9	Insulinoma	Distal pancreatectomy	N/A	N/A	155	10	None (No POPF)	8
Hagendoorn (2018) [21]	Netherlands	Case report	1	10	SPT	Pancreato duodenectomy	Pancreatico jejunostomy	No	NR	NR	DGE (No POPF)	12
Chinnusamy (2018) [23]	India	Case report	1	14	SPT	Pancreato duodenectomy	Pancreatico jejunostomy	No	480	120	None (No POPF)	6
Lalli (2019)[22]	Canada	Case report	1	17	SPT	Distal pancreatectomy	N/A	N/A	277	Nil	None (No POPF)	6
Schulte Am Esch (2021) [19]	Germany	Case report	1	10	Insulinoma	Enucleation	N/A	N/A	110	Minimal	Symptomatic Pseudocyst	11
Nota (2021)[20]	Netherlands	Case report	1	11	pNET	Distal pancreatectomy	N/A	N/A	NR	NR	None (No POPF)	6
van Ramshorst (2021) [18]	Netherlands	Case report	1	16	SPT	Central pancreatectomy	Pancreatico jejunostomy	Internal	248	20	(Grade B POPF)	8
Froehlich (2022) [26]	USA	Case report	1	11	SPT	Enucleation	N/A	N/A	NR	Minimal	None (No POPF)	2
Sergi (2023) [27]	Italy	Case report	1	17	SPT	Distal pancreatectomy	N/A	N/A	410	Nil (0)	Symptomatic fluid collection	16
Bennitt (2025) [28]	USA	Case report	1	11	SPT	Distal pancreatectomy	N/A	N/A	315	10	None (No POPF)	5

Legend: EBL, Estimated Blood Loss; POPF, Postoperative Pancreatic Fistula; DGE, Delayed Gastric Emptying; pNET, Pancreatic Neuroendocrine Tumor; SPT, Solid Pseudopapillary Tumor; LOS, Length of Hospital Stay; NR, Not Reported; N/A, Not Applicable

Among resections requiring reconstruction, a PJ was performed in all three procedures (two PDs and one CP) [18, 21, 23]. No study reported the use of an externalized pancreatic duct stent.

Operative time was reported in 7 studies and ranged from 110 to 480 min, depending on the procedure [18, 19, 22–24, 27, 28]. Estimated blood loss, when reported, was low (median 10 mL, IQR 0–20 mL) [18, 19, 22–24, 26–28]. One patient among those included in the review developed a Grade B POPF, which was managed conservatively with octreotide and antibiotics. This occurred following a CP [18]. The remaining patients for whom data were available had no POPF. One patient developed a symptomatic pancreatic pseudocyst after enucleation [19], one had DGE after PD [21], and another developed a symptomatic fluid collection that required percutaneous drainage (drain fluid amylase not reported) [27]. Median length of hospital stay was 8 days (IQR 6–12 days).

### Technical focus: Roux-en-Y PJ with externalized duct stent

In addition to the systematic review, we provide a detailed technical description of a reproducible surgical approach consisting of robotic CP with Roux-en-Y PJ and externalized pancreatic duct stent placement in two consecutive pediatric patients with SPT. Intraoperative and postoperative details are summarized in Table 2. The complete robotic technique is presented in Supplementary Video 1.

**Table 2:** Intraoperative and postoperative characteristics of our two consecutive cases of robotic central pancreatectomy

	Age	Operative time, (min)	EBL, (mL)	Complications (POPF)	CD complication grade	Drain removal (days)	LOS, (days)	Pathological diagnosis
Case 1	12	386	100	No (BL)	0	9	11	Radically (R0) resected SPT, 30 mm
Case 2	17	350	100	POH (POPF B)	II	13	8	Radically (R0) resected SPT, 18 mm

Legend: EBL, Estimated Blood Loss; POPF, Postoperative Pancreatic Fistula; CD, Clavien-Dindo; LOS, Length of Hospital Stay; BL, biochemical leak; POH, postoperative hyperamylasemia; SPT, Solid Pseudopapillary Tumor

### Case 1 (video case)

**Fig. 2:**
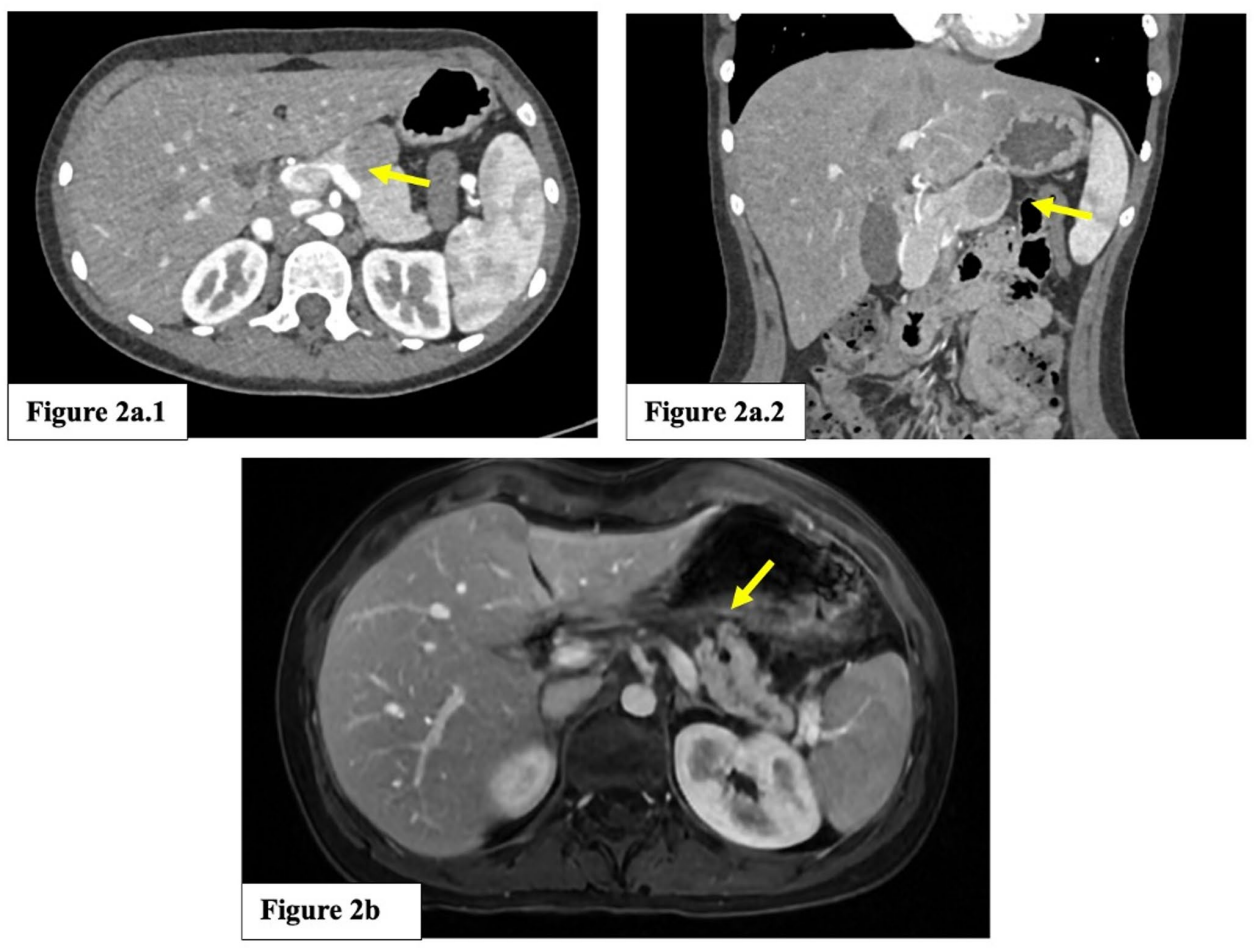
**a**. Preoperative computed tomography (CT) images. (1) Axial view showing the well-circumscribed lesion (arrow). (2) Coronal view demonstrating the relationship with the surrounding pancreatic parenchyma (arrow). **b**. Postoperative control MRI. Axial LAVA image shows the Roux-en-Y Pancreaticojejunostomy (arrow) with preserved pancreatic remnant

A 12-year-old girl with a 30 mm SPT of the pancreatic body underwent robotic CP with end-to-side Roux-en-Y PJ with externalized stent placement. Preoperative computer tomography (CT) images are showed in Fig. 2a. After complete exposure of the pancreas, intraoperative ultrasound was performed to confirm the location of the lesion and to assess its relationship with the splenic vessels, allowing their preservation and accurate planning of the transection lines. The pancreas was carefully mobilized: first, the inferior margin was dissected to identify the splenic vein, then the superior margin was dissected to expose the splenic artery and create a retropancreatic tunnel. Distal and proximal pancreatic transections were performed to ensure adequate parenchymal margins, while maintaining a parenchyma-sparing intent. Distal pancreatic transection was performed using robotic cold scissors with selective parenchymal haemostasis, and proximal transection was completed with an ECHELON^®^ 60 mm (Gold cartridge) stapler.

A Roux limb was prepared (30 cm distal to the ligament of Treitz; divided with stapler) and brought up through a small avascular mesocolic window for reconstruction.

An end-to-side, two-layer duct-to-mucosa PJ was then performed. First, the posterior layer between the posterior pancreatic capsule and the jejunal seromuscular wall was completed with continuous non-absorbable 4 − 0 sutures (Fil-block^®^). Second, a duct-to-mucosa anastomosis was fashioned with interrupted 5 − 0 non-absorbable monofilament stitches. At this stage, an externalized transanastomotic stent (PankreaPlus^®^ polyvinyl catheter; Peter Pflugbeil GmbH^®^) was inserted into the main pancreatic duct, advanced intraluminally within the Roux limb for approximately 10 cm and then exteriorized via a small anterior abdominal stab incision after a short Witzel tunnel. It was then secured with monofilament absorbable sutures to both the pancreatic duct and the jejunal mucosa to prevent migration and ensure decompression of the anastomosis during the healing phase. Third, the anterior layer of the PJ was completed with continuous non-absorbable 4 − 0 sutures (Fil-block^®^), reinforcing the anastomosis and stabilizing the stent.

The specimen was extracted through a Pfannenstiel incision. Finally, two open (Easy-Flow) drains were placed: one adjacent to the pancreatic stump and one posterior to the PJ, before undocking the robot. Operative time was 386 min, and estimated blood loss was 100 mL. Pathologic examination confirmed an R0 resection of SPT, with no evidence of capsular invasion, vascular involvement, or perineural invasion.

The postoperative course was uneventful apart from a biochemical leak (BL). Last drain was removed on postoperative day (POD) 9, the patient was discharged on POD 11, and the externalized transanastomotic duct stent was removed on POD 45, as per internal protocol. At 6-month follow-up, the patient was asymptomatic, with preserved endocrine and exocrine pancreatic function (fasting glucose: 90 mg/dL; fecal elastase: 450 µg/g) and no evidence of recurrence.

### Case 2

**Fig. 3:**
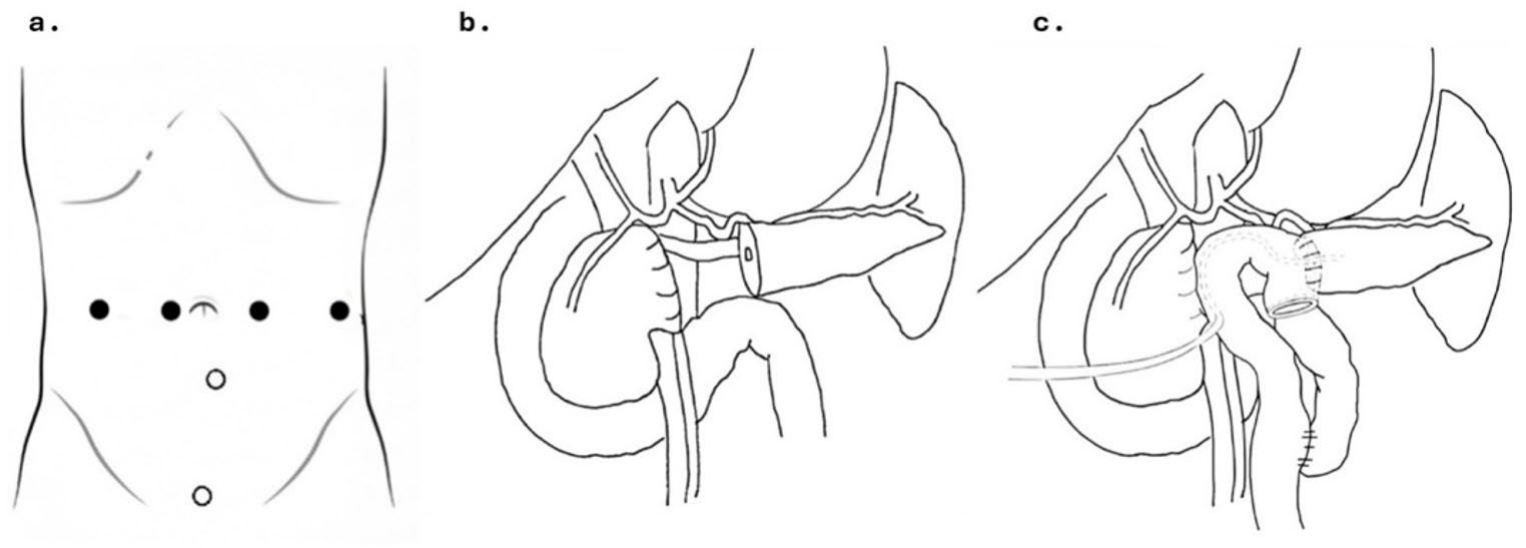
(**a**) Trocar placement schematic: black dots indicate robotic ports; white dots indicate assistant laparoscopic ports. (**b**) Parenchymal transection complete, showing the proximal stapled stump and distal pancreatic remnant. (**c**) Reconstruction phase showing the completed Roux-en-Y Pancreaticojejunostomy with externalized transanastomotic stent

A 17-year-old girl with an 18 mm SPT of the pancreatic neck underwent the same robotic CP with end-to-side Roux-en-Y PJ with externalized stent placement; the overall procedural phases are illustrated in Fig. 3. Operative time was 350 min, and estimated blood loss was 100 mL. Intraoperative and postoperative details are summarized in Table 2.

The postoperative course was characterized by postoperative hyperamylasemia (POH) and grade B POPF, managed conservatively with prolonged drainage, gradually mobilized, and parenteral nutritional support. The patient was discharged on POD 8 with a drain in place. The drain was removed in the outpatient clinic on POD 13, following clinical and biochemical improvement. The externalized transanastomotic duct stent was removed on POD 45, as per internal protocol. At 6-month follow-up, endocrine and exocrine pancreatic function were normal (fasting glucose: 95 mg/dL; fecal elastase: 420 µg/g), with no late complications or recurrence (Fig. 2b).

Pathologic examination confirmed an R0 resection of SPT, with no evidence of capsular invasion, vascular involvement, or perineural invasion.

### Risk-of-bias assessment

Overall methodological quality was limited, with recurrent issues related to case selection, completeness of follow-up, and outcome ascertainment, as expected for single-case designs. Detailed item-level judgements according to JBI checklists are provided in Supplementary section 3.

## Discussion

This systematic review shows that robotic pancreatic resections in pediatric patients remain extremely rare, with only ten single-patient reports identified worldwide [18–24, 26–28]. No prior study has described the use of externalized pancreatic duct stent in CP, despite adult data supporting its ability to decrease the POPF burden [11, 12]. Indeed, in our experience one patient developed a clinically relevant (CR) POPF. As a consequence, although we demonstrated that robotic CP with Roux-en-Y PJ and externalized pancreatic duct stenting is technically feasible in selected pediatric patients, no conclusions can be drawn regarding its effectiveness or potential benefit. In this context, our surgical description is intended to provide technical insight rather than additional clinical evidence.

Emerging pediatric surgical evidence supports the feasibility of robotic procedures in pediatric oncology. In a large single-center experience from Beijing Children’s Hospital, Chang et al. reported 114 consecutive robotic operations —including two pancreatic resections— with an overall postoperative complication rate of 2.6% and median hospital stay of six days, suggesting that robotic surgery can be safely implemented in children within specialized, high-volume centers [9]. These findings are consistent with a large prospective nationwide series by Blanc et al., which included 100 robotic pediatric oncology procedures, indicating that the robotic approach can be selectively used in a multi-institutional context [29]. Nevertheless, pancreatic resections accounted for only a small proportion of procedures in both studies, and CP remained uncommon, reflecting the limited use of parenchyma-sparing strategies in this population [9, 29]. 

The robotic platform, with its 3D visualization and wristed instrumentation, could be particularly suited to addressing the challenges posed by the soft, friable pediatric pancreas, enabling the precise dissection, delicate parenchymal handling, and duct-to-mucosa anastomosis essential for tissue preservation. Despite these advantages, robotic experience in younger patients may be constrained by reduced operative space, which increases the risk of robotic instrument collision. Another concern is the prolonged operative time of robotic pancreatic resections. The operative time of these procedures appear relatively long; however, it should be interpreted in the context of the technical complexity of robotic CP, especially once a Roux-en-Y reconstruction is applied. In our systematic review, operative times reached up to 480 min, and similar durations have been reported in adult robotic pancreatic surgery, particularly for complex resections such as PD [7, 30, 31]. 

The rationale for pursuing a technically demanding procedure with high postoperative morbidity, such as CP, lies in its potential long-term benefits, including preservation of pancreatic function. In addition, in children and adolescents, the ability to offer a less invasive surgical approach, associated with reduced postoperative pain, faster recovery, and a lower aesthetic impact, is of substantial importance, as these factors may significantly influence psychological well-being and overall quality of life.

In the context of benign or low-grade malignant pancreatic lesions, located in the pancreatic neck and body, CP represents an appropriate pancreatic parenchyma- and spleen-sparing strategy. Compared with DP or PD, CP maximizes preservation of endocrine and exocrine tissue while sparing the pancreatic head, the distal pancreas and the spleen, thereby reducing lifelong risks of diabetes, malabsorption, and OPSI [2–5]. However, these benefits come at the cost of higher short-term postoperative morbidity. CP has consistently been associated with higher POPF rates compared with DP, primarily due to the presence of two pancreatic transection surfaces. Indeed, CP combines the risk of a distal pancreatic stump with that of a PJ anastomosis, resulting in a so-called “dual-stump” configuration that contributes to increased morbidity but not mortality [32, 33]. This further reinforces that CP should be reserved for high-volume centers with extensive experience in pediatric-pancreatic surgery with the specialized expertise and resources required to anticipate this increased morbidity risk and manage pancreas-specific complications effectively [34, 35]. However, because CP is performed for benign conditions that do not require adjuvant treatment, these increased morbidity risk may be acceptable in order to preserve a better quality of life in patients with a long life expectancy.

Facing this evidence, the use of an externalized pancreatic duct stent represents a potential mitigation strategy for POPF, providing controlled external drainage of pancreatic juice and mechanical decompression across the anastomosis during the critical healing phase. In our center, this approach is routinely adopted in all pancreatic anastomoses deemed at high risk for POPF—particularly in the presence of a soft pancreatic texture and a small-caliber main pancreatic duct. In adult pancreatic surgery, the role of externalized stenting has been evaluated in high-risk PJ. In a randomized clinical trial by Andrianello et al., externalized stenting was shown to be a valid mitigation strategy to reduce the clinical burden of a POPF [12]. However, its applicability to CP—particularly in pediatric patients—remains uncertain and should not be assumed. In this context, the use of externalized pancreatic duct stent in our cases should be interpreted as a technical adjunct rather than a validated strategy to reduce fistula occurrence. Importantly, the occurrence of Grade B POPF in one of the two patients further emphasizes that no conclusions can be drawn regarding its effectiveness, which requires specific validation.

Given the extremely limited evidence base and the descriptive nature of the available data, several limitations must be acknowledged. The current evidence is limited to case reports with short follow-up, preventing meaningful comparison of functional or long-term oncologic outcomes. The rarity of pediatric pancreatic tumors and the specialized expertise required further limit data collection. Furthermore, the small number and non-comparative design of the included studies precluded meta-analysis and formal assessment of publication bias in this systematic review.

In summary, this systematic review - complemented by two detailed procedural descriptions and video documentation - provides a comprehensive overview of robotic parenchyma-sparing pancreatic surgery in children and adolescents. While our experience provides technical insight, it does not establish effectiveness. Future collaborative multicenter efforts and standardized registries are needed to better define indications, refine technical protocols, and evaluate the role of mitigation strategies such as the externalized pancreatic duct stents in the pediatric CP.

## Conclusion

Robotic pancreatic surgery in pediatric patients remains rare, with the published literature restricted to case reports. This study showed that robotic CP with Roux-en-Y PJ and an externalized transanastomotic pancreatic duct stent may represent a technically feasible pancreas-preserving option in selected pediatric patients with benign or low-grade malignant tumors of the pancreatic body, when performed in experienced high-volume centers. However, given the extremely limited evidence and the absence of comparative data, no conclusions can be drawn regarding its effectiveness or potential benefit in reducing postoperative complications. These findings should be considered hypothesis-generating and require validation in larger, multicenter studies.

## Supplementary Information

Below is the link to the electronic supplementary material.

Supplementary Material 1

Supplementary Material 2

## Data Availability

No datasets were generated or analysed during the current study.
